# Changes in the Serum Fatty Acid Profile After Anhepatic Phase of Orthotopic Liver Transplantation Procedure

**DOI:** 10.3389/fphys.2022.817987

**Published:** 2022-03-29

**Authors:** Aleksandra Hliwa, Adriana Mika, Maciej Sledzinski, Dariusz Laski, Bruno Ramos-Molina, Tomasz Sledzinski

**Affiliations:** ^1^ Department of Pharmaceutical Biochemistry, Faculty of Pharmacy, Medical University of Gdansk, Gdansk, Poland; ^2^ Department of General, Endocrine and Transplant Surgery, Faculty of Medicine, Medical University of Gdansk, Gdansk, Poland; ^3^ Obesity and Metabolism Group, Biomedical Research Institute of Murcia (IMIB-Arrixaca), Murcia, Spain

**Keywords:** fatty acids, liver transplantation, metabolism, cirrhosis, metabolomics

## Abstract

During orthotopic liver transplantation (OLT), the patients’ body remains deprived of this organ for some time, which could cause critical changes in the levels of various metabolites in the circulation, including fatty acids. Thus, the aim of this study was to determine whether the liver transplantation procedure leads to significant changes in the FA profile in serum lipids after the anhepatic phase. Our gas chromatography–mass spectrometry analysis revealed that after transplantation, serum levels of myristic and palmitic acids significantly decreased, whereas serum levels of very long-chain FAs containing 20 or more carbons in their chains were increased. These results indicate that the anhepatic phase during liver transplantation produces significant changes in serum fatty acid levels, and emphasizes the role of the liver in the metabolism of very long-chain fatty acids.

## Introduction

The prevalence of chronic liver diseases, including non-alcoholic fatty liver disease (NAFLD) and alcoholic liver disease (ALD), is increasing worldwide (Lazo and Clark, 2008; Younossi et al., 2015). Whereas excessive alcohol consumption is the most common cause of end-stage liver disease (Osna et al., 2017), non-alcoholic steatohepatitis (NASH) represents the fastest growing indication for liver transplantation in Western countries (Burra et al., 2020). Patients with NASH are at risk of worsening outcomes, such as end-stage liver dysfunction and hepatocellular carcinoma (HCC) (Younossi et al., 2019; Hliwa et al., 2021). Thus, it is estimated that approximately 20% of patients with NASH develop cirrhosis, whereas the risk of HCC in patients with NASH has increased about 8-fold (Younossi et al., 2019).

Orthotopic liver transplantation (OLT) is the treatment of choice for patients with liver failure in end-stage liver disease, being the only chance of survival in many cases. An early detection of liver damage is critical for successful therapy, but unfortunately, most of the end-stage liver disease cases are asymptomatic and only highly invasive methods like liver biopsy can provide an accurate diagnosis of liver disease in these patients. OLT is a high-risk surgical procedure associated with increased mortality and morbidity. Furthermore, it is related to a lifelong need of immunosuppression and all its associated comorbidities. During the transplantation, the patients’ body remains deprived of this organ for some time, called the anhepatic phase, which can cause critical changes in the levels of various metabolites in the blood. Thus, blood collection just before the surgery and at the end of the anhepatic phase provides a unique opportunity to examine the effect of the lack of liver function on the fatty acid (FA) composition in blood, highlighting the physiological role of this organ in FA metabolism.

FAs are essential metabolites in the human body that serve as a source of energy but are also involved in many processes including inflammation, cellular signaling, and regulation of metabolism (Mika and Sledzinski, 2017). Given that the liver is the major organ involved in the synthesis of FAs and that certain advanced hepatic complications are characterized by changes in FA levels, the aim of this study was to determine whether the anhepatic phase of the OLT procedure leads to changes in the FA profile of serum lipids, which may affect the liver recipients’ metabolism during surgery.

## Materials and Methods

### Subjects

Eighteen patients (3 women and 15 men), who underwent orthotopic liver transplantation in the Department of General, Endocrine, and Transplant Surgery at the Medical University of Gdansk, were included in this study. Nine patients were suffering from alcoholic liver disease (ALD), three patients were suffering from cirrhosis associated with HCV or HBV virus infection, one patient was diagnosed with NASH, and the rest of our patients were diagnosed with liver cirrhosis, probably due to NAFLD development. Two patients from this group were using statins. All subjects were qualified for transplantation surgery according to the current clinical standards and transplant procedures (POLTRANSPLANT - http://www.poltransplant.org.pl), due to their end-stage liver dysfunction. Basic laboratory parameters were determined in the Central Clinical Laboratory at the Medical University of Gdansk, as a routine laboratory test before transplantation surgery. Liver transplant recipients were between 19 and 68 years old. The OLT procedure was divided into three phases. A first pre-anhepatic phase in which native liver was exposed and prepared to resection. Native cirrhotic liver was dissected from the surrounding tissues, leaving only the main vascular structures (hepatic veins, portal vein, and hepatic arteries). In the second anhepatic phase, these vessels were clamped and dissected, and then the native liver was completely removed from the abdominal cavity; during this phase, the donor’s liver portal vein is anastomosed and venous outflow is reconstructed (piggyback or classic technique). The clamps were removed, and portal reperfusion of donor’s liver occurred. The anhepatic phase lasted in our transplant center an average of 75 min. During the third neohepatic phase, anastomoses of the hepatic artery and bile duct were performed, completing the whole procedure. During transplantation, patients were under standard, complex anesthesia with tracheal intubation. Anesthesia induction for surgery was performed with sufentanil, propofol, and atracurium. Anesthesia was continued with continuous infusion of sufentanil and atracurium and simultaneous inhalation of desflurane. Intravenous fluids (mostly crystalloids) were given under central venous pressure to maintain hemodynamics and productive diuresis. Inotropic agents (mostly noradrenalin) and blood products (blood, cryoprecipitate, fibrinogen, platelets, and fresh frozen plasma) were given only if clinical situation forced to use them to maintain stable hemodynamics and homeostasis. The protocol of our study was prepared in compliance with the Declaration of Helsinki of the World Medical Association and received an approval from the Local Bioethics Committee at the Medical University of Gdansk (decision no. NKBBN/423/2020-2021). Written consent was obtained from all the subjects, before sample collection.

### Sample Collection

Blood samples were collected just before OLT, approximately 15 min before the patient’s anesthesia was started. A second blood sample was collected during the surgery, 30 min after portal reperfusion (the earliest moment after the end of the anhepatic phase when, for medical reasons, it is possible to collect blood from the patient). All samples were collected in tubes without anticoagulant, kept for 30 min at room temperature for clotting process, and then centrifuged at 3000 x g for 15 min at 4°C. After centrifugation, the serum samples were immediately stored in aliquots at -80°C until analysis.

### Sample Preparation

Total lipids from serum samples were extracted using a mixture of chloroform:methanol 2:1 (v/v) as mentioned in Folch et al. (1957). Next, the chloroform phase was evaporated to dryness under nitrogen stream, and all lipids present in serum were hydrolyzed with a 0.5 M KOH/methanol solution at 90 C. After incubation, 6 M HCl was added to the mixture to neutralize, and FAs were extracted three times in water/n-hexane. The FA methyl esters (FAMEs) were obtained by methylation with 10% BF3 in methanol. FAMEs were triply re-extracted with n-hexane, and dried samples were stored at -20 C until analysis. Chloroform, methanol, HCl, and n-hexane were obtained from Avantor Performance Materials Poland S.A. (cat no. POCH: 234429154, POCH: 621991154, POCH: 575283115, POCH: 466311155). BF3 in 10% methanol was obtained from Sigma-Aldrich, USA (cat. no. 15716).

### Gas Chromatography–Mass Spectrometry Analysis

The analysis of FAMEs was conducted using a GC–EI–MS QP-2010SE (SHIMADZU, Japan) spectrometer with a capillary column of 30 m × 0.25 μm × 0.25 μm (Phenomenex, Torrance, CA, USA). The column was set at 60–310 C, rising 4 C each min, and maintained at 310 C for 5 min; the analysis lasted for 67.5 min. The electron energy for FAME ionization was performed at 70 eV. helium was used as a carrier gas, and the column head pressure was determined at 100 kPa. Moreover, we used full scan mode analysis with the mass scan range set at m/z 45–700 in. The identification of FAs was assisted by using reference standards (37 FAME Mix, Sigma-Aldrich, USA, cat no. CRM47885) and library NIST2021. Then 19-methylarachidic acid (Sigma-Aldrich, USA, cat no. M5531) was used as an internal standard. We used the previously described analytical approach in several previous studies (Pan et al., 1994; Mika et al., 2017; Mika et al., 2020; Mika et al., 2021).

### Data Analysis

The data obtained in the study were presented as mean ± standard deviation (SD). Serum FA levels were presented as mean % (the amount of each specific FA with respect to total FA content). The differences in serum FA levels before and after the surgery were analyzed by using the paired *t*-test, since all those data were normally distributed. A *p* value below 0.05 was considered statistically significant.

## Results

The biochemical characteristics of the study patients are presented in Table 1. The study patients did not show abnormal levels of total cholesterol, LDL, and TAG, but they displayed low HDL levels. In addition, they had high serum levels of liver enzymes (ALT, AST, GGT, and ALP), bilirubin and CRP, whereas the albumin level was below the reference range.

**TABLE 1:** Clinical characteristics of study group before orthotopic liver transplantation (mean ± SD).

Parameter	Value	Reference range
Age (years)	50.9 ± 13.5	—
BMI (kg/m^2^)	28.5 ± 5.26	—
MELD-Na score	16.9 ± 10.3	—
Underlying liver disease		
ALD (n)	9	—
HCV/HBV (n)	3	—
NAFLD/NASH (n)	6	—
Biochemical parameters		
Total cholesterol (mg/dl)	128 ± 51.7	114–190
LDL (mg/dl)	84.9 ± 33.2	<115
HDL (mg/dl)	37.1 ± 15.7	>40
TAG (mg/dl)	72.3 ± 27.8	<150
CRP (mg/L)	13.0 ± 19.5	0.0–0.5
Bilirubin (mg/dl)	4.25 ± 7.26	0.1–1.2
GGT (IU/L)	120 ± 92.5	3–40
ALT (U/L)	91.3 ± 170	5–40
AST (U/L)	81.9 ± 163	5–40
ALP (U/L)	219 ± 381	37–98
Creatinine (mg/dl)	1.35 ± 0.96	0.6–1.3
Albumin (g/L)	33.3 ± 8.38	35–50

BMI, body mass index; LDL, low-density lipoprotein; HDL, high-density lipoprotein; TAG, triacylglycerols; CRP, C-reactive protein; GGT, gamma-glutamyl transpeptidase; ALT, alanine aminotransferase; AST, asparagine aminotransferase; ALP, alkaline phosphatase; ALD, alcoholic liver disease; NAFLD, non-alcoholic liver disease; NASH, non-alcoholic steatohepatitis; HCV, hepatitis C virus, HBV, hepatitis B virus.

a Reference range used by the Central Clinical Laboratory, Medical University of Gdansk.

**FIGURE 1:**
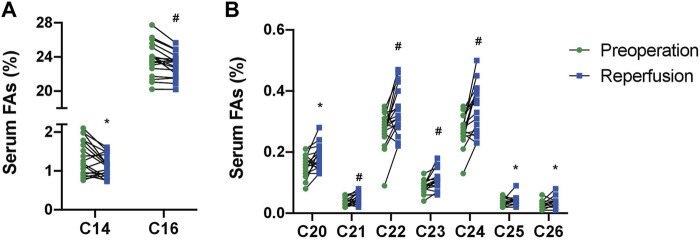
Levels of palmitic (C16), myristic (C14) **(A)**, and very long-chain FAs (C20-C26) **(B)** from serum lipids in patients with end-stage live dysfunction before liver transplantation and after reperfusion (mean % ± SD). *p<0.05; #*p* < 0.001.

The full serum FA profile before the surgery procedure and after liver reperfusion is given in Supplementary Table S1. After reperfusion, no significant changes were found in the serum levels of FAs when classified by subfamilies (Table 2). However, analysis of individual FA species revealed a significantly lower content of two saturated fatty acid (SFA) representatives, namely, 14:0 and 16:0 (Figure 1A). In addition, we detected significantly higher amounts of very long-chain FAs (VLCFAs) in patients’ sera, both from even-chain and odd-chain subgroups, including 20:0, 21:0, and longer FAs—up to 26:0 (Figure 1B).

**TABLE 2:** Levels of FA subfamilies from serum lipids in patients with end-stage liver dysfunction before liver transplantation and after reperfusion (mean % ± SD).

	Before surgery	After reperfusion	P (paired *t*-test)
ECFA	33.3 ± 1.79	32.7 ± 1.87	0.059
OCFA	0.88 ± 0.13	0.90 ± 0.13	0.612
Iso-BCFA	0.18 ± 0.048	0.18 ± 0.048	0.424
Anteiso-BCFA	0.21 ± 0.061	0.22 ± 0.071	0.255
Di-M-BCFA	0.051 ± 0.022	0.052 ± 0.015	0.911
Total BCFA	0.44 ± 0.10	0.45 ± 0.12	0.705
Total SFA	34.6 ± 1.94	34.1 ± 1.99	0.073
MUFA	35.4 ± 6.11	34.5 ± 4.87	0.416
PUFA n6	27.6 ± 6.91	28.8 ± 5.47	0.189
PUFA n3	2.36 ± 0.91	2.51 ± 0.90	0.217

ECFA, even-chain fatty acids; OCFA, odd-chain fatty acids; BCFA, branched-chain fatty acids; MUFA, monounsaturated fatty acids; PUFA, polyunsaturated fatty acids.

## Discussion

Our study revealed significant changes in the FA profiles from serum lipids during the OLT procedure for the first time, providing new evidence about the possible role of liver in FA metabolism during transplantation. One of the most significant changes shortly after the transplantation was a decrease in the serum levels of the SFA species—14:0 and 16:0. This possibly may be due to the cessation of the *de novo* lipogenesis during the anhepatic phase of surgery. 16:0 is the main product of fatty acid synthase (FASN), which uses malonyl-CoA, acetyl-CoA, and NADPH as substrates, and it is the highest activity present in the liver (Mika and Sledzinski, 2017). FASN produces 16:0 predominantly and also small amounts of 14:0. Moreover, 14:0 can also come from a shortening of 16:0, likely by peroxisomal β-oxidation (Rioux et al., 2007). The interruption in the course of the aforementioned processes may explain a decrease in 16:0 and 14:0 after the anhepatic phase of the transplantation procedure. 16:0 is a substrate for further elongation and desaturation to 18:1, and this is a major FA included in TG that is synthesized and released by the liver in the form of very low-density lipoprotein (VLDL) (Mika et al., 2015).

The most relevant changes in serum FA concentrations after reperfusion were observed in VLCFAs. VLCFAs are synthetized by hepatocytes in a multi-step process mediated by FA elongases (Zwara et al., 2021). VLCFAs can be incorporated into other lipid structures, for example, sphingolipids, and they are degraded in peroxisomes. Their fragmentation process occurs mostly in hepatic structures (Stradomska et al., 2013; Wanders, 2014). Thus, we can hypothesize that the increase in their serum levels may be due to the lack of VLCFA degradation in liver peroxisomes during the anhepatic phase. Moreover, it cannot be excluded that increased serum VLCFAs may influence the patients’ health, since their elevated levels have been reported in various pathological conditions. For instance, they are elevated in peroxisomal disorders including Zellweger syndrome or X-linked adrenoleukodystrophy (Engelen et al., 2012; Stradomska et al., 2020) and in colorectal cancer (Mika et al., 2017; Mika et al., 2021). However, whether changes in these FAs during the surgical process have a negative effect on the body requires further investigation.

On the other hand, previous studies have shown that n-3 fatty acids are protective in hepatic ischemia reperfusion injury (Baker et al., 2019). However, in our study, we have not found any significant changes in serum n-3 fatty acids levels during liver transplantation (Table 2).

Other studies on FA alterations in patients with liver diseases showed various FA alterations including elevated monounsaturated fatty acids (MUFAs) (Puri et al., 2007; Hliwa et al., 2021) and SFAs (Wang et al., 2011; Chiappini et al., 2017) in the liver samples of patients with NAFLD. Others reported higher levels of total FFAs, and several SFA and MUFA species in the serum of patients with NAFLD (Puri et al., 2009; Feng et al., 2017; Hliwa et al., 2021). The clinical significance of the changes in the FA profile shortly after transplantation is currently unknown, and our ongoing research, consisting of the follow-up at different time intervals after surgery and comparison with the healthy people, in the larger group of study subjects, will likely allow us to answer this question in the near future. This preliminary study is a rather and unique opportunity to observe the effect of the short-term lack of liver function in the human body on FA metabolism. Thus, our results have a physiological rather than clinical significance and provide novel data on the essential role of the liver in VLCFA metabolism. One limitation of our study was the relatively small number of patients included for analysis. However, despite this limitation, our statistical approach (paired statistical test) revealed significant changes of certain FA species during the OLT procedure, suggesting that the results are convincing. Another limitation of our study is heterogeneity in terms of sex, age, and underlying liver disease. In fact, despite the differences in patient outcomes and some clinical features, ALD and NAFLD are very similar in terms of pathophysiology and histopathological changes, in which fatty liver, steatohepatitis, and cirrhosis are the main symptoms, and often, the allocation of a patient to the NAFLD group consists only denial of alcohol consumption (Tannapfel et al., 2011; Toshikuni et al., 2014). As can be seen in Supplementary Table S2, narrowing the study group by sex, age, and underlying liver disease did not change the general conclusions. The last limitation is that we cannot exclude the effect of anesthesia during surgery on the serum FA profile.

Our study concerns the lipid metabolism in the liver, which is a major site for the regulation of lipid metabolism in the human body. The lipids enter the liver in the form of remnant chylomicrons and LDL particles and then they form endosomal vesicles which are bound with lysosomes, and all those complex lipids (mainly triacylglycerols and phospholipids, and also sphingolipids and cholesterol esters) are hydrolyzed into free fatty acids, glycerol, and other basic compounds. Moreover, some fatty acids enter the liver in its free form (mainly from adipose tissue bound to albumin in the circulation). Thus, fatty acids originated from various complex lipids, and free fatty acids delivered to the liver from the adipose tissue can be built in other complex lipids and transported outside the liver in the form of VLDL particles. Thus, considering that the liver plays a key role in the modulation of serum fatty acid profile, we measured the composition of fatty acids in total lipids without discriminating the lipid fraction. However, although the liver is believed to be the central organ that controls lipid homeostasis, serum FA levels can also be affected by other organs such as adipose tissue to a lesser extent. Our previous study showed that 18:1 was the main FA present in lipids stored in the human adipose tissue (Mika et al., 2015). However, this FA was not altered during OLT, which suggests that the adipose tissue does not play a key role in the observed changes after the surgical intervention. Thus, given that the liver is the main organ affected by the surgical procedure, we postulate that those changes in serum FAs are mainly related to the temporary absence of the liver.

## Conclusion

In conclusion, our results revealed that the anhepatic phase of the liver transplantation procedure led to a significant decrease in the levels of the SFAs—14:0 and 16:0 from serum lipids and an increase in VLCFAs, possibly due to a break in the functioning of the liver during this surgical phase. The fact that even relatively short break in the functioning of the liver causes changes in serum levels of the aforementioned FAs suggests a major role of the liver in 14:0, 16:0, and VLCFA metabolism. Further studies will be conducted to monitor the serum FA profiles at longer times after OLT in transplanted patients.

## Data Availability

The original contributions presented in the study are included in the article/Supplementary Material, further inquiries can be directed to the corresponding author.
