# Supplementary material for: Changes in the Serum Fatty Acid Profile After Anhepatic Phase of Orthotopic Liver Transplantation Procedure
Source: Front Physiol. 2022 Mar 29;13:817987. doi: 10.3389/fphys.2022.817987 (PMC9004627; doi:10.3389/fphys.2022.817987)
Supplement: Supplementary file 1 [file Table1.DOCX]

Changes in the serum fatty acid profile during anhepatic phase of orthotopic liver transplantation procedure

Aleksandra Hliwa, Adriana Mika, Maciej Sledzinski, Dariusz Laski, Bruno Ramos-Molina and Tomasz Sledzinski

**Table S1. Serum fatty acid profile in patients just before liver transplantation and just after reperfusion (serum; mean % ± SD)**

| **FA** | **Before LT** | **After reperfusion** | **P (paired T-test)** |
| --- | --- | --- | --- |
| 12:0 | 0.19 ± 0.16 | 0.13 ± 0.071 | 0.107 |
| 13:0 | 0.022 ± 0.010 | 0.015 ± 0.005 | 0.019 |
| 14:0 | 1.34 ± 0.45 | 1.14 ± 0.28 | 0.025 |
| 15:0 | 0.38 ± 0.097 | 0.37 ± 0.068 | 0.595 |
| 16:0 | 23.7 ± 1.96 | 23.1 ± 1.44 | 0.004 |
| 17:0 | 0.29 ± 0.048 | 0.28 ± 0.038 | 0.236 |
| 18:0 | 7.21 ± 1.03 | 7.60 ± 0.85 | 0.238 |
| 19:0 | 0.027 ± 0.005 | 0.032 ± 0.011 | 0.020 |
| 20:0 | 0.16 ± 0.036 | 0.18 ± 0.041 | 0.011 |
| 21:0 | 0.036 ± 0.014 | 0.050 ± 0.016 | 0.001 |
| 22:0 | 0.27 ± 0.059 | 0.34 ± 0.076 | 0.003 |
| 23:0 | 0.087 ± 0.019 | 0.109 ± 0.031 | 0.002 |
| 24:0 | 0.28 ± 0.052 | 0.35 ± 0.079 | 0.001 |
| 25:0 | 0.033 ± 0.012 | 0.042 ± 0.014 | 0.031 |
| 26:0 | 0.028 ± 0.012 | 0.036 ± 0.016 | 0.049 |
| 28:0 | 0.009 ± 0.006 | 0.009 ± 0.007 | 0.334 |
| iso 12-M-13:0 | 0.013 ± 0.005 | 0.014 ± 0.007 | 0.772 |
| iso 13-M-14:0 | 0.026 ± 0.010 | 0.030 ± 0.013 | 0.168 |
| iso 14-M-15:0 | 0.047 ± 0.016 | 0.044 ± 0.015 | 0.384 |
| iso 15-M-16:0 | 0.083 ± 0.030 | 0.076 ± 0.024 | 0.151 |
| iso20-M-21:0 | 0.012 ± 0.007 | 0.014 ± 0.005 | 0.260 |
| anteiso 12-M-14:0 | 0.059 ± 0.020 | 0.066 ± 0.018 | 0.144 |
| anteiso 14-M-16:0 | 0.10 ± 0.036 | 0.10 ± 0.039 | 0.476 |
| anteiso 16-M-18:0 | 0.040 ± 0.017 | 0.037 ± 0.022 | 0.660 |
| anteiso20-M-22:0 | 0.010 ± 0.005 | 0.015 ± 0.006 | 0.003 |
| 2,6,10-M-12:0 | 0.026 ± 0.010 | 0.028 ± 0.009 | 0.263 |
| 4,8,12-M-14:0 | 0.026 ± 0.015 | 0.024 ± 0.010 | 0.703 |
| 14:1 | 0.085 ± 0.067 | 0.059 ± 0.034 | 0.091 |
| 16:1 | 4.78 ± 2.04 | 4.25 ± 0.92 | 0.156 |
| 18:1 | 30.1 ± 4.35 | 30.1 ± 4.36 | 0.946 |
| 19:1 | 0.014 ± 0.005 | 0.018 ± 0.006 | 0.015 |
| 20:1 | 0.19 ± 0.056 | 0.20 ± 0.060 | 0.675 |
| 22:1 | 0.034 ± 0.018 | 0.028 ± 0.009 | 0.271 |
| 24:1 | 0.33 ± 0.069 | 0.29 ± 0.060 | 0.019 |
| CPOA2H | 0.11 ± 0.041 | 0.10 ± 0.031 | 0.095 |
| 16:2n6 | 0.011 ± 0.003 | 0.012 ± 0.005 | 0.668 |
| LA (18:2n6) | 21.7 ± 5.01 | 22.0 ± 4.13 | 0.651 |
| ARA (20:4n6) | 4.43 ± 1.69 | 4.77 ± 1.71 | 0.170 |
| DGLA (20:3n6) | 1.10 ± 0.44 | 1.18 ± 0.43 | 0.255 |
| 20:2n6 | 0.15 ± 0.037 | 0.14 ± 0.031 | 0.669 |
| AdA (22:4n6) | 0.14 ± 0.037 | 0.14 ± 0.033 | 0.811 |
| ALA (18:3n3) | 0.30 ± 0.13 | 0.31 ± 0.13 | 0.601 |
| EPA (20:5n3) | 0.68 ± 0.38 | 0.73 ± 0.39 | 0.186 |
| ETA (20:4n3) | 0.062 ± 0.019 | 0.064 ± 0.017 | 0.532 |
| DHA (22:6n3) | 1.01 ± 0.44 | 1.05 ± 0.44 | 0.542 |
| DPA (22:5n3) | 0.30 ± 0.091 | 0.31 ± 0.088 | 0.648 |

Linoleic acid – LA (18:2n6), arachidonic acid – ARA (20:4n6), dihomo-γ-linolenic acid – DGLA (20:3n6), eicosadienoic acid (20:2n6), adrenic acid - AdA (22:4n6), α-linolenic acid – ALA (18:3n3), eicosapentaenoic acid - EPA (20:5n3), eicosatetraenoic acid - ETA (20:4n3), docosahexaenoic acid – DHA (22:6n3) and docosapentaenoic acid – DPA (22:5n3)
