# Supplementary material for: Changes in the Serum Fatty Acid Profile After Anhepatic Phase of Orthotopic Liver Transplantation Procedure
Source: Front Physiol. 2022 Mar 29;13:817987. doi: 10.3389/fphys.2022.817987 (PMC9004627; doi:10.3389/fphys.2022.817987)
Supplement: Supplementary file 2 [file Table2.DOCX]

Supplementary Table 2. Serum fatty acids in healthy controls and subgroups of OLT patients with the same sex (males), limited age range (35-64) and specified underlying liver disease (ALD or NASH/NAFLD).

| Fatty acid | All OLT patients (n=18) | | Male OLT patients (n=15) | | OLT patients aged 35 – 64 years (n=14) | | OLT patients with ALD (n=9) | | OLT patients with NASH/NAFLD (n=6) | |
| --- | --- | --- | --- | --- | --- | --- | --- | --- | --- | --- |
|  | BEFORE | AFTER | BEFORE | AFTER | BEFORE | AFTER | BEFORE | AFTER | BEFORE | AFTER |
| 14:0 | 1.34 ± 0.45 | 1.14 ± 0.28* | 1.25± 0.42 | 1.08 ± 0.28* | 1.39 ± 0.46 | 1.16 ± 0.31* | 1.26 ± 0.42 | 0.998 ± 0.20 | 1.47 ± 0.54 | 1.27 ± 0.29 |
| 16:0 | 23.7 ± 1.96 | 23.1 ± 1.44* | 23.6 ± 1.90 | 22.9 ± 1.46* | 24.0 ± 1.77 | 23.3 ± 1.25* | 23.6 ± 1.27 | 23.0 ± 1.0* | 23.9 ± 2.76 | 23.1 ± 1.93 |
| 20:0 | 0.16 ± 0.036 | 0.18 ± 0.041* | 0.16 ± 0.036 | 0.18 ± 0.046 | 0.16 ± 0.039 | 0.19 ± 0.042* | 0.17 ± 0.034 | 0.19 ± 0.044 | 0.14 ± 0.039 | 0.16 ± 0.032 |
| 22:0 | 0.27 ± 0.059 | 0.34 ± 0.076* | 0.27 ± 0.062 | 0.33 ± 0.076* | 0.28 ± 0.065 | 0.35 ± 0.08* | 0.28 ± 0.038 | 0.35 ± 0.083 | 0.26 ± 0.090 | 0.32 ± 0.082 |
| 24:0 | 0.28 ± 0.052 | 0.35 ± 0.079* | 0.27 ± 0.056 | 0.34 ± 0.079* | 0.28 ± 0.058 | 0.35 ± 0.077* | 0.28 ± 0.039 | 0.36 ± 0.085* | 0.27 ± 0.077 | 0.33 ± 0.082 |
| 26:0 | 0.028 ± 0.01 | 0.036 ± 0.016 | 0.026 ± 0.009 | 0.036 ± 0.018* | 0.03 ± 0.01 | 0.039 ± 0.016 | 0.029 ± 0.006 | 0.039 ± 0.017 | 0.028 ± 0.019 | 0.028 ± 0.012 |

* - p<0.05
